# Achievement of Therapeutic Goals with Low-Dose Imiglucerase in Gaucher Disease: A Single-Center Experience

**DOI:** 10.1155/2013/151506

**Published:** 2013-10-28

**Authors:** Irina Tukan, Irith Hadas-Halpern, Gheona Altarescu, Ayala Abrahamov, Deborah Elstein, Ari Zimran

**Affiliations:** ^1^Shaare Zedek Medical Center, Affiliated to the Hadassah-Hebrew University School of Medicine, Ein Karem 91031, Israel; ^2^Gaucher Clinic and Preimplantation Genetics Unit, Shaare Zedek Medical Center, Affiliated to the Hadassah-Hebrew University School of Medicine, Ein Karem 91031, Israel

## Abstract

Gaucher disease, a lysosomal storage disorder, is a multisystem disorder with variable and unpredictable onset and severity. Disease-specific enzyme replacement therapy (ERT) has been shown to reverse or ameliorate disease-specific hepatosplenomegaly and anemia and thrombocytopenia. ERT also impacts bone manifestations, including bone crises, bone pain, and appearance of new osteonecrosis, and improves bone mineral density to varying degrees. The objective of this study was to assess achievement of predefined therapeutic goals based on international registry outcomes for Israeli patients with Gaucher disease receiving imiglucerase for four consecutive years on a low-dose regimen followed in a single center. All data were taken from patient files. The therapeutic goals were taken from standards published in the literature for disease-specific clinical parameters. Among 164 patients at baseline, values for spleen and liver volumes, hemoglobin and platelet counts, and Z-scores for lumbar spine and femoral were significantly different from the goal. After four years ERT, there was a significant improvement (*P* = 0.000) in each of the therapeutic goal parameters from baseline. 15.2% of these patients achieved all hematology-visceral goals. In children, there was achievement of linear growth and puberty. This survey highlights the good overall response in symptomatic patients receiving low-dose ERT with imiglucerase in Israel.

## 1. Introduction

Gaucher disease, a prevalent lysosomal storage disorder, is a multisystem disorder with variable and unpredictable onset and severity especially in the common nonneuronopathic form [1]. Patients with nonneuronopathic (type 1) Gaucher disease may suffer from varying degrees of splenomegaly, hepatomegaly, thrombocytopenia, bleeding tendencies, anemia, hypermetabolism, skeletal pathology, growth retardation in children, and/or pulmonary disease; invariably, health-related quality of life is affected. Age of onset may be in any decade of life, and there are no apparent triggers to onset; yet there are also periods of quiescence even in patients with severe manifestations such as debilitating and irreversible skeletal complications. Disease characteristics, including genotype, provide only a statistical estimation of the expected trajectory of the signs and symptoms of the disease for any particular patient. 

Disease-specific enzyme replacement therapy (ERT) with mannose-terminated glucocerebrosidase (alglucerase, Ceredase, Genzyme Corporation, Cambridge, MA, USA) was introduced in 1991 [2]. Subsequently, a recombinant enzyme (imiglucerase, Cerezyme, Genzyme Corporation, Cambridge, MA) superseded the placental product [3, 4] and has been shown to reverse or ameliorate many of the visceral and hematological manifestations of Gaucher disease. ERT also impacts bone manifestations, including bone crises, bone pain, and appearance of new osteonecrosis in the joints and pathological fractures, and improves bone mineral density (BMD) to varying degrees [5]. However, due to the variable patterns of onset, progression, and severity, the initiation of treatment and the evaluation of the therapeutic response have often been controversial [6]. 

In 2004, a group of international experts in Gaucher disease [7] defined a set of therapeutic goals for the primary parameters of Gaucher disease that would benchmark successful interventions. These “goals” reflected the experience recorded for ERT with imiglucerase and as such also provided a means to assess alternative therapies and modalities as they enter the clinical arena. The goals as described at that time were derived from a decade of experience and data from more than 3000 patients treated with imiglucerase as reported to the International Collaborative Gaucher Group (ICGG); the registry is supported by the Genzyme Corporation (now a Sanofi company). Specifically, the achieved therapeutic response, respectively, in the realms of anemia, thrombocytopenia, hepatomegaly, splenomegaly, (four features of) skeletal pathology, and health-related quality of life (HRQoL) following a minimum of four years of imiglucerase exposure was equal to the therapeutic goal for that feature. However, monitoring these therapeutic goal achievements has proved difficult even among registry cohorts because more than half the patients evaluated had incomplete/partial clinical responses relative to these therapeutic goals [8]. Nonetheless, among the findings [8] garnered from these studies is that the higher-dose regimen (60 units/kg body weight/every-other-week) achieves a more rapid response relative to that achieved with lower doses (15 units/kg body weight/every-other-week) in the key disease-specific parameters of anemia, thrombocytopenia, and spleen and liver sizes; also see [3].

The purpose of the current study was to assess achievement of the therapeutic goals for patients receiving imiglucerase for four consecutive years on a constant low-dose regimen which is the standard in Israel for symptomatic Gaucher disease [9].

## 2. Methods

Our local institutional review board (Helsinki Committee) deemed that patient consent was not required for this retrospective analysis.

Beginning in 1992 [4], all patients who initiated ERT with imiglucerase were included if there was a complete data set from advent of ERT and after approximately 4 years of uninterrupted exposure to imiglucerase and at a constant dosage of 15 units/kg body weight/every-other-week for most of the adults and 30/kg body weight/every-other-week for most of the children (<14 years at start of ERT and <18 years at end of study). These dosing regimens are those that are standard for all patients who have been approved for ERT by the Gaucher Committee of the Israeli National Ministry of Health.

All data were taken from patient files. Laboratory tests were performed in the central hematology laboratory of the Shaare Zedek Medical Center. Spleen and liver volumes were performed by ultrasonography by a single senior radiologist (IH-H) using the three long axes with the organ volume considered a multiplicand of these [10]. 

Complaints of bone pain at any site were culled from the patient files.

Evidence of new osteonecrosis was based on radiological evidence in comparison to the full plain X-ray set taken at presentation in all patients; new complaints and/or functional changes were indications for complaint-specific radiological work-up in any patient at any time. 

Bone mineral density (BMD) and Z-scores were performed using dual energy X-ray absorptiometry (DXA; Hologic, Bedford, MA, USA) at the lumbar spine (LS) and femoral neck (FN) according to the manufacturer's instructions.

The therapeutic goals were taken from Pastores et al. [7] for the clinical parameters. For pediatric patients who entered the study at age <14 years, achievement of puberty and (pediatric) height was also included.

Since our clinic does not provide tools of health-related quality of life as part of the annual follow-up protocol, this feature of the therapeutic goals is not included.

### 2.1. Statistical Analysis

Descriptive statistics were used and one-sample (two-tailed) Student's *t*-test was applied to ascertain whether goals were achieved after four years of ERT. A significance level of 5% was predetermined. Logistic regressions with start and end measures as independent and/or predictors were employed when controlling for the possible confounders and/or proposed predictors of gender, age, and splenic status to control for these factors that might impact attainment of the therapeutic goal.

## 3. Results

There were 164 patients who met the criteria for inclusion; that is, they had been receiving a constant dose for approximately four years from advent of ERT. 

There were 42 patients for whom there was documentation about puberty and/or pediatric height while on ERT (ages 4–14 years at start of study). 

Of all patients, 65 patients (39.6%) were male; the mean age was 39.7 years, range 8–78 years, at end of the four years. There were 59 patients (36%) who were homozygous for the N370S mutations, and the rest had various compound heterozygous genotypes but not all with one N370S allele. There were 35 patients (21.3%) who were splenectomized. 

Table 1 presents the mean values at baseline and after four years of ERT with imiglucerase and the percent achieving respective therapeutic goals.  Table 2 provides the therapeutic goals as per Pastores et al. [7] for comparison as to the expectations regarding values that are “at goal” at presentation or at any point thereafter. According to these criteria, not all disease-specific parameters are expected to normalize within four years, and hence comparison to local normal values was not employed. Importantly, <10% were at goal for anemia at baseline, and in addition, despite the fact that 21% of patients were splenectomized, the mean platelet counts at baseline was below the lower limit of the normal range. The mean BMD Z-scores for LS and FN were both in the negative range with the mean LS Z-score in the osteopenic range; despite the fact that children and splenectomized patients were included, only 70% were at goal at baseline for BMD Z-scores.

At baseline, values for the four hematology-visceral parameters and BMD Z-scores for LS and FN were significantly different than the goal as per Table 2. At the end of four years, there was a significant improvement (*P* = 0.000) in each of the parameters from baseline; between 60% and 94% of patients achieved the respective goals at four years. 15.2% of all the patients achieved therapeutic goals in both hematological and visceral parameters within 4 years.

For the pediatric patients, because not all the children had achieved the age of sexual maturation at the end of the study, then, as to be expected, only some of them achieved the goal (albeit statistically significant for the group). Similarly, for these children, but also for the young adults who had not achieved the age of peak bone density (25–30 years), the goal was improvement in the absolute value of BMD (as versus improvement in T-scores which were not used in this study for this reason), and this was statistically significant after four years for the cohort. 

Table 3 shows achievement of therapeutic goals after four years in the current cohort on low-dose (mean = 34.2 units/kg/4 weeks) imiglucerase. Table 3 also shows the differences in percentages from baseline to four years where possible. The current cohort had 35 patients (21.3%) who were splenectomized at baseline and would be expected to have high-normal platelet counts: but only 37 patients (22.6%) had platelet counts >150,000/mm^3^ at baseline, and not all of these were the splenectomized patients. 

For the goal of BMD (at both LS and FN), only an improvement in absolute values was considered as achieving the therapeutic goal (see above); however, for the respective Z-scores, achieving a normal value of 0 or better (non-negative score) was considered as achieving the therapeutic goal. 

When logistic regression was applied to ascertain the effect of gender, age, or spleen status on each of the hematological (hemoglobin and platelet counts) and visceral (spleen and liver size), the only significant effect was on liver size by gender (*P* = 0.013) and age (*P* = 0.008) where achievement of the goal of decreasing liver size was less in males and in younger patients, respectively. However, when applying analysis of variance (ANOVA) post hoc with the therapeutic goal of each of the above four parameters, respectively, as a dependent variable and the other variables constant (gender, genotype, age, and parameter values at start and end), all were significant (*P* = 0.000). 

## 4. Discussion

With more than two decades of experience with imiglucerase, there is abundant evidence of its effectiveness in amelioration of the disease-specific parameters of Gaucher disease. Nonetheless, the issue of dosage has never been resolved in a practical sense. In 1995, the seminal papers on safety and efficacy of imiglucerase in the context of two clinical trials were published, which, respectively, showed approximately equivalent efficacy of the recombinant enzyme imiglucerase relative to placenta-derived alglucerase at 60 units/kg/infusion [3] and equivalent efficacy at low-frequency (every-other-week) relative to high frequency (three times a week) in a low-dose (15 units/kg/infusion) regimen [4]. While the manufacturer (Genzyme Corporation, Cambridge, MA, USA) originally recommended the high-dose regimen, countries with limited health budgets were not uncomfortable to adopt the low-dose regimen. Nonetheless, it was also obvious that higher dosing regimens were able to achieve better numerical values than those achieved at lower doses. Indeed, by cognizance of these findings and despite the fact that the starting regimens for ERT in Israel are 15 units/kg and 30 units/kg biweekly for adults and children, respectively, in the past there had always been recourse to the Israeli National Gaucher Committee to request doubling of the dose for those patients whose response was deemed inadequate by the treating physician. Nonetheless, with regard to the responses to imiglucerase based on a recent analysis of ICGG data [11] that demonstrated differences in response in favor of higher dosages, the actual clinical significance of those differences in patients who may have responded well to lower doses (albeit more slowly) is debatable [12].

However, along with acknowledging the importance of inducing a rapid response in patients with life-threatening symptoms and signs, there is an appreciation of the actual symptoms and signs of most patients with type 1 Gaucher disease at presentation which are not life-threatening. It is the case in many of the wealthier countries that all identified patients with Gaucher disease are treated, and mostly at the high-dose regimen. This is dramatically different in less wealthy countries such as Israel where only symptomatic patients who meet the disease-specific severity criteria of a national Gaucher Committee are eligible for governmental reimbursement/support of this expensive therapy. This is clearly evident in the baseline characteristics of the current cohort versus that of Weinreb et al. [8] (which also includes some patients from our clinic). The overall implication from the current data as a stand-alone report about the effect of low-dose regimen in achieving the standard therapeutic goals is that even in very symptomatic patients there will be a beneficial response within four years. The percent of patients achieving those goals is not greatly different than those benchmarked by Weinreb et al. [8]. Although some may contend that the quality of these comparisons (of achievement of goals for disease-specific parameters and also for the nonparametric markers) is imperfect, making interpretation difficult, in that the Gaucher community has come to accept the Pastores et al. criteria as a benchmark of therapeutic response, we are comfortable with these outcomes using low-dose regimens. 

The means for all parameters including pediatric height and achievement of puberty were significantly lower than expected at baseline based on goals to achieve improved status and/or normalization. This underscores the fact that the patients in this cohort were approved for ERT by virtue of comparable objective criteria; that is, they presented with these same criteria in order to be eligible for ERT. In all parameters, there was a significant increase in mean values from baseline to four years. It is also noteworthy that the mean platelet counts were below the normal range at baseline despite a large percentage of splenectomized patients. This reflects the fact that for some patients who initiated ERT many years after splenectomy (most often in childhood in the era before ERT), platelet counts did not remain in the upper normal range, and the progressive decrease in counts was a continuing marker of disease severity. 

Regarding achievement of age-specific goals in the pediatric patients, it is important to note that, in a report from the ICGG of the effect of imiglucerase on children [13], median height Z-score was −1.4 at baseline but required eight years of ERT to approximate the median value for the normal population. In the current cohort, virtually all the children, who were at least 18 years at the end of the 4-year study period, had achieved midparental height within four years.

Drilling down to the outcomes presented here for the entire cohort, one notes that genotype was not predictive of achieving any of the goals, despite the fact that there was a majority of patients who were compound heterozygotes (and not necessarily with even one N370S allele). Age and gender impacted achieving goals only for liver size for which this may be an idiosyncratic finding and for which we have no clinical comparator.

Limitations of this study are that it is both retrospective and dependent on patients for whom there were all the parameters at nearly exactly four years from the advent of ERT. This paradigm disallowed those who do not come for annual followups, are not in Israel at the fourth annual followup, and those who either did or did not achieve the goals at a point earlier than four years but were recruited to clinical trials for switchover to a different ERT. 

Another limitation is the issue of compliance with the ERT regimen because it was based on patients' claim of compliance. 

The most important limitation of the study is that the evaluation of the therapeutic goals was at four years when the Pastores et al. [7] timeline for some of the goals is within one or two years. However, this limitation can be explained as derivative from the objectives of the study because the results in the current cohort were intended to also be compared to those of Weinreb et al. [8] who also used an approximately four-year interval. 

## 5. Conclusions

In conclusion, this retrospective look at the achievement of therapeutic goals in a single Israeli center using low-dose imiglucerase in a real-life setting relative to what has heretofore been reported vis a vis the achievement of these goals at a mean of higher doses highlights the good overall response to imiglucerase in symptomatic patients. The issue of the clinical relevance of achieving goals has been raised with regard to the goal of improving hemoglobin because in Gaucher disease it has been shown to be correlated with the risk of avascular necrosis [14]. The goal for hemoglobin was achieved within four years in this cohort. It is also assumed that the remaining goals were achieved at a comparable timeline to the cohort at a higher mean ERT doses and that most patients approached improvement if not having actually achieved normalization of all parameters. The differential in responses among patients with Gaucher disease may be due to several reasons including (1) signs that are considered disease-specific but are actually multi-factorial such as bone pain; (2) differences in target organs/tissues before advent of ERT such as infarcted spleens and/or nodular livers that are poorly responsive to ERT [15]; (3) idiosyncratic events such as pregnancies or viral infections that impact hematological parameters and/or splenomegaly at the time of analysis; (4) associated diseases, being related or unrelated to Gaucher disease; and (5) adverse reactions to ERT whether as a hypersensitivity reaction or antibody formation that in some cases reduce efficacy and/or timeliness of response. Nonetheless, ERT with imiglucerase at a low-dose regimen benefits patients with respect to the predetermined therapeutic goals.

## Figures and Tables

**Table 1:** Baseline values for surveyed parameters and after 4 years (*n* = 164), *P* value between value of difference at four years from baseline, and percent of patients achieving goal for that parameter at four years (Student's *t*-test and logistic regression with significance level of 5% were predetermined.). Therapeutic goals are as described by Pastores et al. (2004) [7] and as presented in Table 2.

	Hemoglobin (gm/dL)	Platelet counts (×10/mm^3^)	Spleen (US) index volume (*n* = 129)	Liver (US) index volume	Bone pain	New bone crises	New osteonecrosis	LS BMD	LS *Z*-score (*n* = 114)	FN BMD	FN *Z*-score (*n* = 112)	Pediatric height	Puberty
Mean ± SD baseline	10.95 ± 1.77	125.61 ± 85.00	2595.18 ± 2423.57	5259.95 ± 3048.00	0.57 ± 0.50	0.09 ± 0.28	0.22 ± 0.41	1.00 ± 0.13	[−1.48] ± 1.43	1.02 ± 0.19	[−0.92] ± 1.88	101.57 ± 24.15	0.00
Mean ± SD 4 years	12.72 ± 1.74	177.01 ± 100.86	1645.43 ± 1356.71	4119.49 ± 1571.65	0.38 ± 0.49	0.02 ± 0.16	0.06 ± 0.24	1.06 ± 0.07	[−1.13] ± 1.18	0.98 ± 0.19	[−0.80] ± 1.61	145.74 ± 22.33	0.51 ± 0.51
*P* value	0.000	0.000	0.000	0.000	0.000	0.000	0.000	0.000	0.000	0.000	0.000	0.000	0.000
% at goal at 4 years	81.7%	68.9%	60.0%	53.1%	59.9%	93.8%	92.6%	NA	69.9%	NA	70.6%	100%	48.8%

US: ultrasound; NA: not applicable.

**Table 2:** Long-term goals (2–5 years) for adults (Pastores et al., 2004 [7]).

Anemia	Women: increasing hemoglobin concentration to ≥11 g/dL;
	Men: increasing hemoglobin concentration to ≥12 g/dL;
	Maintaining improved hemoglobin value achieved in first 1-2 years

Thrombocytopenia	Moderate thrombocytopenia at baseline (60–120 × 10^9^/L): Increase platelet count 1.5 to 2 times by 1 Year, and approach low normal platelet count values by 2 Year.
	Severe thrombocytopenia at baseline (<60 × 10^9^/L): increasing platelet count 1.5 times by 1 Year and continuing to improve platelet counts slightly (2 times by 2 Year)

Splenomegaly	Decreasing spleen volume by 30–50% within 1 Year; reducing and maintaining spleen volume to ≤2–8 times normal or decreasing volume by 50–60% by 2–5 Years

Hepatomegaly	Decreasing liver volume by 20–30% within 1-2 Years; reducing and maintaining liver volume to 1.0 to 1.5 times normal or reducing liver volume by 30–40% by 3–5 Years

Bone pain	Lessening or eliminating bone pain in 1-2 years

Bone crises	Preventing bone crises

Osteonecrosis	Preventing osteonecrosis and joint collapse

Skeletal pathology	Improving trabecular BMD by 3–5 years

**Table 3:** Percent of patients achieving therapeutic goals in 4 years.

Therapeutic goal	Current study (*n* = 164)	Change in 4 years
Anemia	9.1% to 81.7%	72.6%
Thrombocytopenia	20.7% to 68.9%	48.2%
Splenomegaly	60.0% at 4 years	ND
Hepatomegaly	53.1% at 4 years	ND
No bone pain	42.1% to 59.9%	17.8%
No bone crises	91.5% to 93.8%	2.3%
No osteonecrosis	78.7% to 92.6%	13.9%
LS Z-score	69.9% at 4 years	ND
FN Z-score	70.6% at 4 years	ND

*ND: not done.
